# Payment reform and integrated care: the need for evaluation

**DOI:** 10.5334/ijic.1515

**Published:** 2013-12-23

**Authors:** Jeroen N. Struijs

**Affiliations:** Health Care Policy and Practice, Department of Health Policy and Management, Harvard School of Public Health, Boston, Massachusetts, USA

Countries face the twin challenge of providing high-quality care while keeping care systems affordable and accessible during a time of financial crisis and low economic growth. In many countries, payment reforms are seen as a major lever to achieve these challenges. The rationale behind payment reforms emerges from the recognition that the current funding systems are a key cause of care fragmentation leading to suboptimal outcomes in terms of cost and quality.

Payment reform policies have been multifaceted. In several countries payment reforms have utilised bundled payments and/or new capitated funding models to incentivise providers to work collaboratively in provider networks. Some of these payment reforms have resulted in the introduction of new provider-led integrated care organisations such as Accountable Care Organizations in the USA and Care Groups in the Netherlands. In these countries, payers (partially) shift economic risks towards these provider-led organisations who assume a degree of financial and clinical accountability, and are as such responsible for the coordination between care providers and the delivery of care, and collaboration between care providers. Next to the establishment of provider-led entities, these payment reforms also influenced the health care delivery process. For instance, these payment reforms resulted into task reallocation, new innovative disease management programmes and care pathways, and stimulated the uptake of case management potentially supported by telehealth and telecare.

Up until now, insight into the effects of newly introduced payment models is limited or lacking. An explanation may be that the current methodological approaches are not sophisticated enough to disentangle the influence of payment reform from the wider components and influences involved in health care reform more generally. Hence, attribution to the potential benefits (or side effects) of payment reform is problematic. Evaluating the effects of reforms is further complicated by the interaction with differing national, regional and local contextual factors. Given such complexity, rigorous evaluations of the impact of payment reforms are needed before up scaling them. These evaluations require (new) methodologies, which disentangle the effects of the core elements of the payment reform, the core elements of the provider-led entities and the core elements of the health care delivery transformations. Only then, insight in generic and context-specific elements of these multifaceted reforms can be gained, resulting in transferable ‘lessons learned’ for other countries.

Some of the articles in this issue of International Journal of Integrated Care provide insight into the impact of payment reforms on both the quality of care delivered and on providers’ experiences. Whilst these help to build the evidence-base, such studies only partially address the above-mentioned challenges of evaluation. The study of de Bruin et al. [1], for example, evaluated whether diabetes type 2 patients with comorbidity experienced more ‘care gaps’ as compared to diabetes type 2 patients without comorbidity, when participating in a single-disease integrated care programme within Dutch Care Groups. The finding that there are hardly any differences in quality of care between both patient groups implies that these provider-led organisations paid by single-disease bundled payments and programmes are able to ensure quality of care for patients with co-morbidity. These insights are important since Dutch Care Groups assume clinical and financial responsibility for all assigned diabetes patients, including those with co-morbidity.

Another example is the study of Tol et al. [2] who performed a survey among dieticians working as subcontractors within Dutch Care Groups. By doing so, this study is the first to specifically focus on the experiences of care providers subcontracted by those provider-led entities. In the Dutch Care Groups, often exclusively owned by the general practitioners, dieticians must collaborate with general practitioners who also subcontract them for their services. The study clearly describes the critical tensions faced by Dutch dieticians between collaboration and competition within these payment reforms. The changing role of general practitioners also resulted in a conflict of interest since, as both commissioners and providers, the study revealed unintended negative tension between them and the subcontracted dieticians, negatively affecting the collaboration between them.

In addition, the in-depth case study of Maria Lluch [3] provides insight into the role of incentives and reimbursement schemes in the development of integrated care and the uptake of information and communication technologies. In this study, it is suggested that incentives that align social, primary and hospital care are rare. Hence, there is a need to design new payment paradigms in order to stimulate integrated care.

The lack of evidence and evaluation into payment reform as a lever to promote integrated care and more sustainable care systems is a major gap in our knowledge. In the coming years, the International Foundation for Integrated Care and the International Journal of Integrated Care must strive to serve as a central and authoritative voice in the international debate on payment reforms by stimulating learning, education and knowledge exchange between providers, policy makers and scientists. By bringing together the worlds of practice, science and policy, International Foundation for Integrated Care can create a strong ‘platform’ dedicated to achieve sustainable high-quality and more integrated care systems.
